# 

**Published:** 1895-05-04

**Authors:** 


PRICE TWOPENCE.

No. 449, Vol. XVIII, May 4th, 1895.

[Registered at the General Post Office as a Newspaper.

WITH EXTRA NURSING SUPPLEMENT.

Per Annum, 10s. 6d., post free.

Monthly Parts, 6d.; post free, 8d.

Ditto per Annum, 8s. 6d., post free.

### THE HOSPITAL

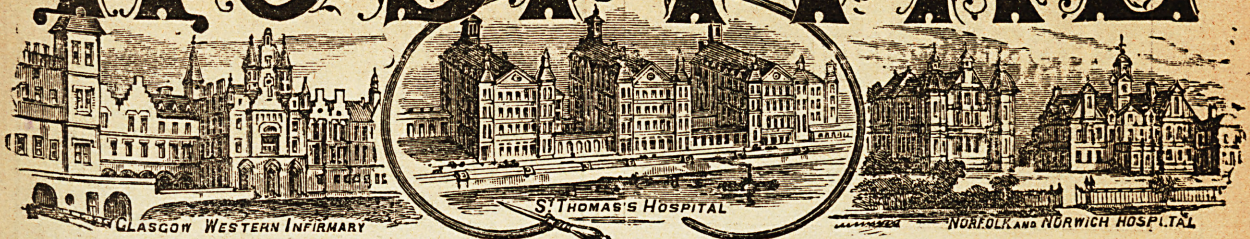

No. 449. Vol. XVIII. WITH EXTRA NURSING SUPPLEMENT. SATURDAY, MAY 4TH, 1895.

